# Primary Care Clinician Perspectives on Older Adult Chronic Pain Management and Clinical Decision Support: Qualitative Study

**DOI:** 10.2196/74381

**Published:** 2025-08-26

**Authors:** Isra Hasnain, Erin M Staab, Ainur Kagarmanova, Marissa Mackiewicz, Mim Ari, Katherine Thompson, Danielle Lazar, Anne Zhao, Glyn Elwyn, Christopher A Harle, Valerie G Press, Neda Laiteerapong

**Affiliations:** 1 Pritzker School of Medicine, Biological Sciences Division, The University of Chicago, Chicago, IL, United States; 2 Section of General Internal Medicine, Biological Sciences Division, Department of Medicine, The University of Chicago, 5841 S Maryland Ave, Chicago, IL, 60637, United States, 1 7738342238; 3 Section of Hospital Medicine, Biological Sciences Division, Department of Medicine, The University of Chicago, Chicago, IL, United States; 4 Section of Geriatrics and Palliative Medicine, Biological Sciences Division, Department of Medicine, The University of Chicago, Chicago, IL, United States; 5 Access Community Health Network, Chicago, IL, United States; 6 The Dartmouth Institute for Health Policy and Clinical Practice, Dartmouth College, Lebanon, United States; 7 Department of Health Policy and Management, Richard M Fairbanks School of Public Health, Indiana University, Indianapolis, United States; 8 Department of Psychiatry and Behavioral Neuroscience, Biological Sciences Division, The University of Chicago, Chicago, IL, United States

**Keywords:** chronic pain, older adults, clinical decision support, opioid use, primary care

## Abstract

**Background:**

Chronic pain management in older adults can be challenging for primary care clinicians due to comorbidities, side effects, and complicated guideline recommendations. Clinical decision support systems (CDSSs) may improve care by integrating guideline-based recommendations, synthesizing relevant patient data, and facilitating shared decision-making. I-COPE (Improving Chicago Older Adult Opioid and Pain Management through Patient-centered Clinical Decision Support and Project ECHO) is an electronic health record–based CDSS designed to gather patient-reported data and support primary care clinicians in managing chronic pain, opioid use, and opioid use disorder in older adults.

**Objective:**

This study examined clinicians’ views on challenges in managing chronic pain and their opinions on I-COPE.

**Methods:**

We conducted semi-structured interviews with 18 clinicians (16 physicians and 2 advanced practice nurses) from 2 University of Chicago Medicine primary care clinics (internal medicine and geriatrics) piloting the I-COPE CDSSs in 2021. The interview guide was informed by the Consolidated Framework for Implementation Research and explored current practices in chronic pain management, challenges, and feedback on I-COPE tools.

**Results:**

Of the 18 participants, 12 (67%) identified as female, 13 (72%) as White, and 9 (50%) had practiced for 10 years or less. Participants stressed the importance of a comprehensive, patient-centered approach to chronic pain management and prioritized multimodal and nonpharmacological treatments. Major barriers to effective chronic pain management were comorbidities, limited visit time, insurance coverage restrictions, and opioid misuse concerns. Most clinicians found the CDSSs beneficial for standardizing multimodal care discussions, enhancing visit efficiency, eliciting patient goals, and facilitating shared decision-making conversations. Clinicians raised concerns about the complexity of the intervention, anticipated issues with clinic workflow, and desired more adaptability. The primary care clinicians in this study demonstrated strong alignment with current pain management guidelines, prioritizing patient-centered pain management using multimodal treatments. They identified I-COPE as a promising tool to reinforce evidence-based practices, increase efficiency, and strengthen patient-clinician communication. However, implementation challenges—particularly around accessibility for older adults, workflow integration, and tool complexity—highlight the need for further refinement and support.

**Conclusions:**

I-COPE offers a promising approach to support primary care clinicians in providing patient-centered guideline-based chronic pain and opioid management for older adults. Further efforts to improve usability and adaptability for real-world workflows and equitable access for older adults should be prioritized.

## Introduction

Clinical guidelines by organizations such as the American Geriatrics Society and Centers for Disease Control and Prevention offer guidance on how to manage chronic pain, opioid use, and opioid use disorder (OUD) in older adults [1,2]. These guidelines stress the importance of understanding each patient’s medical history, past treatments, social situation, and ability to access treatments, as well as using multimodal interventions to manage pain. However, integrating these clinical factors and treatment options in decision-making can be challenging in clinical practice.

Previous work has focused on barriers to chronic pain and opioid management in older adults, including limited clinician time and resources [3,4]. One potential solution to addressing the complexity of chronic pain management in older adults is the use of clinical decision support systems (CDSSs). CDSSs may increase clinician adherence to guidelines by collecting and organizing patient data and presenting conversation aids when necessary. Although CDSSs have been proven effective in various clinical settings, some clinicians have been hesitant to use them due to their complexity [5,6]. If designed well, CDSSs could help address clinician-identified barriers such as lack of access to resources [7] and medical complexity of chronic pain in older adults [8]. Primary care clinicians’ (PCCs) perspectives on the use of CDSSs in chronic pain and opioid use management remain understudied, especially for older adults and when assessing patient health outcomes [9-13].

I-COPE (Improving Chicago Older Adult Opioid and Pain Management through Patient-centered Clinical Decision Support and Project ECHO) was designed for older adult patients (aged 65 years and older) with chronic pain diagnoses, chronic opioid use, or OUD. I-COPE includes a pre-visit patient questionnaire that PCCs can review to enable shared decision-making, alongside clinical decision support, to create a comprehensive pain, opioid, and OUD management plan. I-COPE was developed with experts in geriatrics, palliative medicine, primary care, clinical informatics, shared decision-making, medical education, addiction medicine, behavioral medicine, implementation science, and clinical research. I-COPE was piloted at 2 clinic sites in June 2021 and later implemented at 35 clinics across metropolitan Chicago. This study focused on PCCs’ views on the current state of chronic pain management in older adults and their opinions on the I-COPE CDSS to improve chronic pain, opioid use, and OUD management in this population.

## Methods

### I-COPE Intervention Development

Two University of Chicago Medicine clinics, 1 internal medicine and 1 geriatrics, serving a diverse patient population on the South Side of Chicago, piloted I-COPE starting in June 2021. I-COPE is an electronic health record (EHR)–based toolkit designed in Epic to support the management of chronic pain, opioid use, and OUD in older adults in primary care settings. Details about the intervention and trial design have been previously published [14]. In brief, the I-COPE intervention included four main components (Multimedia Appendices 1-4): (1) a pre-visit questionnaire for patients aged 65 years or older with a history of chronic pain diagnoses, high pain scores during their most recent clinic visit, recent opioid medications, or OUD; (2) an EHR-embedded order set (SmartSet) that included patient education materials, prefilled medications, recommended referrals, and community resource lists; (3) a shared decision-making conversation tool for pain management treatment options; and (4) tailored patient education tools. The questionnaire included validated screeners about current levels of pain (Pain, Enjoyment of Life, General Activity Scale), depression (Patient Health Questionnaire 2), and drug use (single-question drug use screener) [15-17]. The I-COPE questionnaire also asked patients to identify functional goals that might be achieved through better pain management and treatment preferences ranging from self-management to surgical intervention. Both clinics did not have previous clinic-level experience with similar interventions.

### Interview Guide Development

The interview guide (Multimedia Appendix 5) was developed with experts in internal medicine, geriatrics, and qualitative research. The qualitative interviews had 2 primary aims. The first aim was to assess clinicians’ beliefs and perspectives on the management of chronic pain, opioid use, and OUD in older adults. We asked about clinicians’ current practices, including how they assess pain and OUD, discuss treatment goals, decide on a chronic pain treatment plan, prescribe opioid medications, and provide patient education on chronic pain. We also queried clinicians about challenges and barriers to older adult chronic pain management. The second aim was to gauge clinicians’ perspectives on I-COPE. Participants were shown screenshots of the I-COPE questionnaire, I-COPE SmartSet, conversation tool, and a sample of patient education materials developed for I-COPE. They were asked about the anticipated benefits and challenges of using these tools (Multimedia Appendix 6). Clinicians were also asked to reflect on the I-COPE intervention and offer their impressions on how it would fit within their clinical practice. Questions about the I-COPE tools were developed using the intervention and individual characteristics domains of the Consolidated Framework for Implementation Research [18].

### Participant Recruitment

Clinicians were recruited from the 2 I-COPE pilot sites. Eligible participants included attending physicians and advanced practice nurses who were actively practicing at 1 of the 2 sites during the pilot. Recruitment emails describing the study purpose, eligibility criteria, and time commitment were sent by a study team member to all eligible clinicians via institutional email lists. Clinicians who expressed interest were scheduled for individual interviews via Zoom (Zoom Video Communications, Inc). Participation was voluntary. Recruitment and data collection occurred in July 2021 shortly after the I-COPE pilot implementation started.

### Conducting and Analyzing Interviews

One-on-one semistructured interviews lasting up to 45 minutes were conducted by a trained medical student (IH). All interviews were conducted over Zoom videoconferencing, audio-recorded with verbal consent from each participant, and transcribed for analysis. Recruitment continued until theme saturation was reached. IH, AK, ES, and NL determined saturation based on no further accumulation of novel information within any domain for 3 consecutive interviews. Each transcript was coded independently by at least 2 researchers using a template approach [19]. Differences were resolved, and themes were developed through discussion.

### Ethical Considerations

This study was reviewed and approved by the University of Chicago Biological Sciences Division’s institutional review board (IRB20-1580). All participants provided verbal informed consent prior to the start of the interviews. Transcripts were deidentified prior to analysis. Participants were compensated for their time with US $40 electronic gift cards.

## Results

### Overview

Interviews were conducted with 18 clinicians from the internal medicine practice and 1 clinician from the geriatric practice. Most interviewees were physicians (n=16, 89%); the remainder (n=2, 11%) were advanced practice nurses (Table 1). About three-quarters self-identified as White (n=13, 72%), with the remaining participants identifying as Asian (n=3, 17%), African American (n=1, 6%), and multiracial (n=1, 6%). Twelve clinicians self-identified as female (67%) and 6 as male (33%). Half of the clinicians had practiced for 10 years or fewer (9, 50%).

Major themes are described in the following sections: “Current Practices for Chronic Pain Management in Older Adults,” “Barriers to Chronic Pain Management in Older Adults,” and “Anticipated Effects of I-COPE.”

**Table 1.** Demographic and professional characteristics of primary care clinicians interviewed following pilot implementation of the I-COPE (Improving Chicago Older Adult Opioid and Pain Management through Patient-centered Clinical Decision Support and Project ECHO) clinical decision support system for chronic pain and opioid management in older adults.

Characteristics	Values, n (%)
Sex	
Male	6 (33)
Female	12 (67)
Race	
White	13 (72)
Asian	3 (17)
African American	1 (6)
Multiracial	1 (6)
Years in practice	
0‐9	8 (44)
10‐19	4 (22)
20‐29	3 (17)
30+	3 (17)
Clinician type	
Advanced practice nurse	2 (11)
Primary care physician	16 (89)

### Current Practices for Chronic Pain Management in Older Adults

Participants emphasized a comprehensive approach to chronic pain management, favoring multimodal and nonpharmacological treatments while considering patient-specific factors but faced challenges with current pain assessment tools and a lack of standardized patient education (Table 2).

Chronic pain management is within the scope of primary care. Clinicians described chronic pain management as an appropriate and critical issue for primary care because of their longitudinal relationship with patients, highlighting that “the continuity that we have with patients and being able to see the big picture is really helpful” (Participant 1). Some clinicians indicated that “it falls to the primary care doctor to try to do the best they can, to pull together resources and improve their own knowledge and skills, to take care of their patients with chronic pain” (Participant 3).

**Table 2.** Primary care clinician perspectives on current practices in chronic pain management for older adults.

Theme	Quotes
Chronic pain management is within the scope of primary care	“[Pain specialists] tend to offer [patients] a couple of procedures, but no like ongoing counseling and support. So, it falls to the primary care doctor to try to do the best they can, to pull together resources and improve their own knowledge and skills, to take care of their patients with chronic pain” (Participant 3).
Prioritizing multimodal and nonpharmacological treatment	“Maximizing non-pharmacologic approaches both first and, if pain is moderate or severe, thinking about it as a component in addition to pharmacologic therapies” (Participant 9). “The opioids I use are really a last step. I think obviously they’re good pain medications, but I think you have to target them in the right patients” (Participant 9).
Older adult–specific considerations when prescribing medications	“If it’s something that’s limited to a joint or two, I try topical agents to the extent that I can because I’m not only thinking about helping them to mitigate their pain, but also some of the unintended consequences of using something like a non-steroidal” (Participant 11). “If they’re really requiring opioids and they happen to live alone and they’re at risk for a fall, are there different ways that we can manage pain or perhaps manage some things at night versus during the day” (Participant 11).
Inadequacy of numerical pain scale for pain assessment	“If it’s just a one question self-report, there’s a lot of subjectivity in it and it’s harder to get an accurate sense of the person’s pain… what does a five even mean” (Participant 2). “I find the most important question when talking to people about chronic pain is a functional evaluation” (Participant 1).
Nonstandard approach to patient education	“I’ve got a great app that I use that gives good information on medication interactions and what available evidence is out there because it’s not stuff I would necessarily read” (Participant 9).

#### Prioritizing Multimodal Treatment

In line with clinical guidelines, clinicians prioritized multimodal strategies and “maximizing nonpharmacologic approaches” (Participant 9). Clinicians said that they often referred patients to physical therapy, which they saw as a valuable component of multimodal treatment. In general, clinicians said that they rarely initiated opioids but instead mostly continued opioid medications prescribed by a prior clinician and used opioids as a “last step” after failure of several nonopioid options (Participant 9).

#### Older Adult–Specific Considerations When Prescribing Medications

When selecting medications, clinicians considered a wide range of factors for older adults. For example, topical analgesics were often reported as the first-line therapy because of their relative safety, especially for patients with comorbidities to avoid “some of the unintended consequences of using something like a nonsteroidal [anti-inflammatory drug]” (Participant 11). Clinicians also considered the risk of adverse effects and patient circumstances; one clinician reported using different approaches to “manage some things at night versus during the day” depending on whether the patient lives alone or is at risk for falls (Participant 11).

#### Inadequacy of Numerical Pain Scale for Pain Assessment

Some participants were dissatisfied with using a numeric pain scale (eg, 0-10 visual analog scale) because of its oversimplification and did not find it clinically useful due to “a lot of subjectivity” (Participant 2). Participants expressed a desire for a more nuanced pain assessment to better understand the impact of chronic pain on patients’ lives and conduct “a functional evaluation” (Participant 1).

#### Nonstandard Approach to Patient Education

Several clinicians desired more readily accessible educational materials, especially for patients with lower health literacy. They reported needing a standardized approach to educate patients about pain management. Some provided verbal education; a few drew visuals to explain concepts or provided patient education via handouts or electronically. Some clinicians were concerned that if they did offer education, patients would not remember what was discussed or review the materials they shared.

### Barriers to Chronic Pain Management in Older Adults

Participants described several challenges to managing chronic pain in older adults, including medical complexity and competing priorities, insurance restrictions, and concerns about opioid misuse (Table 3).

**Table 3.** Primary care clinician–identified barriers to chronic pain management in older adults.

Theme	Quotes
Medical complexity and competing priorities	“People are not coming in for their pain only, they’re coming in with...ten other medical problems. So that’s the challenge, what’s the priority?” (Participant 10). “It’s a little easier in a younger, healthier person, who doesn’t have like kidney disease, or ulcers, or is confused by multiple meds…It’s like the multi-morbid, complex patient that kind of gets overwhelming” (Participant 3). “We just don’t have that capability sometimes of having multiple visits with our patients given my patient volume load” (Participant 17). “There are a lot of folks with chronic pain, where they’re far more worried about their shortness of breath. Or, you know, quitting smoking or why is the hemoglobin falling? Again, what you’re hearing is competing demands on bandwidth for both the patient and the provider” (Participant 12).
Restrictive insurance coverage	“I’d love to maximize things like topical diclofenac or topical lidocaine…with patients that have Medicare, there’s often difficulty in getting coverage for those medications” (Participant 9). “We can offer these things, but they need to be reimbursed. And for a lot of our patients, it’s just not practical that they will be able to do these things out-of-pocket. It’s just not in their budget to pay for their own acupuncture” (Participant 1). “It’s hard because I feel like chronic pain at every visit is like the same thing. Like I don’t have a lot more to offer, you know, if they tried physical therapy, they tried injections…I’m a little limited in my options of what to do for them” (Participant 17). “A lot of the over-the-counter pain medications, our patients can’t afford, and insurance won’t pay for. The insurance company will pay to give them [acetaminophen/hydrocodone], but they won’t pay for you to give them lidocaine patches” (Participant 1).
Concerns about opioid misuse	“I do feel mean, by not increasing their opioids. You’re always like, ‘Am I being an uncaring physician? Or am I being a good, responsible steward of opioids?’” (Participant 3). “I think everybody that practices outpatient medicine or inpatient medicine has experiences where you have patients that are struggling with OUD or some other overlay, which complicates chronic pain management” (Participant 5). “You want to give people the benefit of the doubt, but at the same time we know that diversion is happening…at what point does a suspicion actually feel warranted as opposed to questioning myself, ‘Am I discriminating against this patient? Am I being biased?’” (Participant 15). “Access to behavioral medicine and substance [use] counseling is very limited. So, if you think someone is overusing, or misusing their opioids, there’s not a lot of options” (Participant 3).

#### Medical Complexity and Competing Priorities

Clinicians commented on having limited options due to comorbidities and potential side effects of Beer’s criteria medications, including nonsteroidal anti-inflammatory drugs and opioids; one clinician shared that “[caring for] multi-morbid, complex patient[s] kind of gets overwhelming” (Participant 3). Clinicians mentioned having insufficient time during visits to address all of patients’ health concerns and how more frequent visits were not always feasible due to “patient volume” (Participant 17). Thus, managing chronic pain amidst other medical concerns and priorities was a challenge due to “competing demands on bandwidth for both the patient and the provider” (Participant 12).

#### Restrictive Insurance Coverage

Some clinicians could not prescribe their desired treatment plan due to insurance restrictions, especially for topical analgesics that “patients can’t afford” (Participant 1). Access to over-the-counter medications and other options, such as acupuncture, was limited since “it’s just not practical that patients will be able to do these things out-of-pocket” (Participant 1).

#### Concerns About Opioid Misuse

Participants shared that opioid misuse and OUD “complicate chronic pain management” (Participant 5). Some clinicians were concerned about prescribing opioids amidst the opioid epidemic while also wanting to give their patients “the benefit of the doubt” (Participant 15). Furthermore, clinicians stated that “access to behavioral medicine and substance [use] counseling is very limited” for patients with OUD (Participant 3).

### Anticipated Effects of I-COPE

#### Overall Intervention

Participants shared that I-COPE tools could “increase the likelihood” of talking about multimodal care (Participant 9) by prompting both clinicians and patients to consider a diverse range of treatment options, fostering a more comprehensive and nuanced approach to pain management. They also thought that the I-COPE tools could “improve efficiency” (Participant 2) of clinic visits by identifying pain-related issues ahead of time and allowing for more focused discussions. Participants said that a standardized approach could “help a lot of people feel more comfortable about managing chronic pain” (Participant 1) and that improved access to tools and resources in the EHR could increase clinicians’ self-efficacy. Clinicians also hoped that being able to show patients that “this is the clinic’s policy, and this is how everybody does it here” (Participant 2) would foster trust by reducing perceived subjectivity and discrimination and promoting a collaborative, nonadversarial approach focused on improving the patient’s quality of life.

Participants were worried about the intervention’s complexity and the potential “lack of completeness” as some clinicians might use only “bits and pieces” as opposed to the whole intervention (Participant 3). Another concern was raised about the compatibility of I-COPE with existing workflows where multiple health concerns are addressed during 1 visit, highlighting that the I-COPE intervention seems to be built for “a world where the pain diagnosis is the focus of the visit, and often it is not” (Participant 12).

#### Patient Pre-visit Questionnaire

Clinicians appreciated that systematic implementation of this questionnaire would help elicit patient functional goals and preferences and give patients an opportunity to “to talk about pain more than we give them time to talk about it” and “get it down on paper” (Participant 4).

Patient’s discomfort with technology, particularly among older patients who may “not even know how to use their patient portal” (Participant 13), was a concern, prompting clinicians to anticipate a preference for traditional paper-based methods. At the same time, clinicians worried about the additional workload for medical assistants if the questionnaire was administered in the clinic and whether the questionnaire could be reliably completed before patient encounters.

#### Shared Decision-Making Conversation Tool

Participants described the conversation tool, the 1-page document that lists evidence-based examples of pain management strategies for older adults, as patient-centered and “well-suited” to guide conversations based on patient preferences (Participant 5). Clinicians thought that the conversation tool illustrated the multimodal approach to pain management and could help patients understand all the options for their care and “empower them” (Participant 18).

Participants expressed a desire for greater adaptability and personalization of the conversation tool. They said some visits called for more targeted and selective approaches and would prefer to “just discuss the most likely best fit treatments for the patient” to save time (Participant 6). Some clinicians expressed discomfort with offering certain treatments or presenting treatments that “you’ve already had the discussion about it’s not really indicated for your type of pain” (Participant 18).

#### Smart Order Set

Clinicians thought that the SmartSet, “being able to see everything on one screen” (Participant 8), would help them provide better care for their patients. Participants also appreciated having educational materials easily accessible to activate and engage “without having to hand type it each time or start from scratch” (Participant 6).

Participants noted that the SmartSet contains “a lot of text” (Participant 2), which might make it difficult to use in real-world practice. They also mentioned that the electronic format might not accommodate diverse practice styles, as some clinicians prefer using “pen and paper” and avoid “looking at the screen” during visits (Participant 2).

## Discussion

### Background

Chronic pain in older adults is often undertreated and nonoptimally managed. CDSSs offer a promising technology-based solution to assist PCCs in chronic pain management. This qualitative study contributes to understanding how CDSSs can improve chronic pain management in older adults while highlighting the barriers and unmet needs faced by PCCs.

 Our qualitative analysis of clinician interviews yielded several major findings: PCCs (1) tried to prioritize nonpharmacological and multimodal treatment approaches with special considerations for their older adult patients, (2) identified several barriers to effectively managing older adult patients’ chronic pain, and (3) were optimistic about potential benefits of implementation and usage of CDSSs but had reservations and concerns about the practicality of regular use.

### Principal Results

Our findings underscore the importance of a multimodal and multidisciplinary approach to chronic pain management. Clinicians emphasized the value of nonpharmacological adjunct therapies, aligning with the Centers for Disease Control and Prevention’s Guideline for Prescribing Opioids for Chronic Pain [1]. This guideline advocates for a combination of pharmacologic and nonpharmacologic treatments to manage chronic pain effectively. Clinicians were enthusiastic about integrating topical agents, physical therapy, and high-quality patient education tools into treatment plans and appreciated the CDSSs as an effective aid for staying compliant with existing clinical guidelines.  

Clinicians’ frustrations with current practices—such as time constraints and inadequate pain screening tools—highlight the potential of CDSSs to address these issues. Previous studies have demonstrated similar practical challenges [13]. CDSSs can integrate up-to-date knowledge and robust clinical evidence into practice, helping prevent misapplication of guidelines, undertreatment of pain, and overreliance on opioids [20]. Additionally, by presenting patient-specific information to clinicians, CDSSs can assist in managing polypharmacy, highlighting more and less safe medication options and facilitating individualized patient care. To address limited time, one potential strategy is to have clinic visits specifically focused on pain management, rather than appending pain management to preventive and chronic care management visits. Although these visits could leverage the full potential of CDSSs, this approach may not be feasible due to high patient volumes and reimbursement limitations.

Clinicians viewed I-COPE as a valuable tool for improving pain management in older adults through its structured approach to assessment and treatment. This is in line with previous research suggesting that standardized, multimodal interventions can improve clinical efficiency, clinician confidence, and patient outcomes by facilitating more consistent and collaborative care [21,22]. The benefits anticipated by clinicians reflect the broader evidence that well-implemented chronic care models can significantly enhance the quality of chronic pain management. Our findings suggest that the use of pain-focused CDSSs, such as I-COPE, could be enhanced by integrating mechanisms to identify patients’ desire to prioritize pain management ahead of their visits, such as through pre-visit questionnaires or scheduling prompts.

However, clinicians also expressed concerns about the challenges of integrating I-COPE into routine practice, particularly regarding workflow disruption, increased workload, and data completeness. These challenges are well documented in the literature on CDSSs, where multicomponent interventions often face resistance due to their complexity [23]. Future innovations in CDSSs, such as through the use of artificial intelligence, may help synthesize complex patient information and deliver more pointed recommendations to clinicians while preserving clinicians’ decision-making autonomy [24]. Additionally, issues related to technology accessibility among older adults highlight the need for careful planning to ensure equitable implementation, as older populations often encounter barriers to using electronic health tools [25]. Addressing these challenges is crucial for I-COPE, or any CDSS, to achieve its full potential. Technologies that read questionnaires aloud to patients and allow them to respond verbally are increasingly usable and may help reduce this barrier in the future [26].

 While the results of this study provide a valuable contribution to the literature around CDSSs and pain management in older adults in particular, future work is needed. Future research should explore strategies for streamlining the user interface, minimizing documentation burden, and optimizing integration with clinical encounters. Additionally, studies should evaluate how pre-visit tools can be adapted to better accommodate patients with limited digital literacy.

### Limitations

Our study includes several limitations. As is the nature of qualitative analysis, our results are influenced by the researchers’ perceptions. In addition, our sample is limited to a single health system and geographic location. PCCs self-selected to participate in interviews. It is possible that clinicians who participated were more interested in chronic pain care or more engaged with the intervention than those who did not participate, and their experiences, barriers, and facilitators may differ. The small sample size and specificity of the content also make our findings difficult to generalize.

### Conclusions

The findings underscore clinician perspectives on current pain management in older adults and the potential of the I-COPE intervention to address existing barriers. Successful implementation will hinge upon addressing challenges related to workflow integration, clinician and patient adaptation, and accessibility. These insights are crucial for optimizing the delivery of comprehensive pain care and enhancing patient outcomes within primary care settings for older adults.

## Supplementary material

10.2196/74381 Multimedia Appendix 1 I-COPE (Improving Chicago Older Adult Opioid and Pain Management through Patient-centered Clinical Decision Support and Project ECHO) pre-visit patient questionnaire.

10.2196/74381 Multimedia Appendix 2 I-COPE (Improving Chicago Older Adult Opioid and Pain Management through Patient-centered Clinical Decision Support and Project ECHO) electronic order set.

10.2196/74381 Multimedia Appendix 3 I-COPE (Improving Chicago Older Adult Opioid and Pain Management through Patient-centered Clinical Decision Support and Project ECHO) conversation tool.

10.2196/74381 Multimedia Appendix 4 Sample of I-COPE (Improving Chicago Older Adult Opioid and Pain Management through Patient-centered Clinical Decision Support and Project ECHO) patient education materials.

10.2196/74381 Multimedia Appendix 5 Interview guide.

10.2196/74381 Multimedia Appendix 6 Anticipated benefits and challenges of using I-COPE (Improving Chicago Older Adult Opioid and Pain Management through Patient-centered Clinical Decision Support and Project ECHO) intervention.

## Abbreviations

CDSS: clinical decision support system
EHR: electronic health record
I-COPE: Improving Chicago Older Adult Opioid and Pain Management through Patient-centered Clinical Decision Support and Project ECHO
OUD: opioid use disorder
PCC: primary care clinician

## References

[1] Dowell D, Ragan KR, Jones CM, Baldwin GT, Chou R (2022). CDC clinical practice guideline for prescribing opioids for pain—United States, 2022. MMWR Recomm Rep.

[2] American Geriatrics Society Panel on the Pharmacological Management of Persistent Pain in Older Persons (2009). Pharmacological Management of Persistent Pain in Older Persons. J Am Geriatr Soc.

[3] Zomahoun HTV, Visca R, George N, Ahmed S (2021). Effectiveness and harms of clinical decision support systems for referral within chronic pain practice: protocol for a systematic review and meta-analysis. Syst Rev.

[4] Dahlhamer J, Lucas J, Zelaya C (2018). Prevalence of chronic pain and high-impact chronic pain among adults—United States, 2016. MMWR Morb Mortal Wkly Rep.

[5] Huang KTL, Blazey-Martin D, Chandler D, Wurcel A, Gillis J, Tishler J (2019). A multicomponent intervention to improve adherence to opioid prescribing and monitoring guidelines in primary care. J Opioid Manag.

[6] Wang EJ, Helgesen R, Johr CR, Lacko HS, Ashburn MA, Merkel PA (2021). Targeted program in an academic rheumatology practice to improve compliance with opioid prescribing guidelines for the treatment of chronic pain. Arthritis Care Res (Hoboken).

[7] Frost MC, Soyer EM, Achtmeyer CE (2023). Treating opioid use disorder in veterans with co-occurring substance use: a qualitative study with buprenorphine providers in primary care, mental health, and pain settings. Addict Sci Clin Pract.

[8] Kaseweter K, Nazemi M, Gregoire N, Louw WF, Walsh Z, Holtzman S (2023). Physician perspectives on chronic pain management: barriers and the use of eHealth in the COVID-19 era. BMC Health Serv Res.

[9] Spithoff S, Mathieson S, Sullivan F (2020). Clinical decision support systems for opioid prescribing for chronic non-cancer pain in primary care: a scoping review. J Am Board Fam Med.

[10] Nair KM, Malaeekeh R, Schabort I, Taenzer P, Radhakrishnan A, Guenter D (2015). A clinical decision support system for chronic pain management in primary care: usability testing and its relevance. J Innov Health Inform.

[11] Harle CA, Apathy NC, Cook RL (2018). Information needs and requirements for decision support in primary care: an analysis of chronic pain care. AMIA Annu Symp Proc.

[12] Allen KS, Danielson EC, Downs SM (2022). Evaluating a prototype clinical decision support tool for chronic pain treatment in primary care. Appl Clin Inform.

[13] Cuadros P, McCord E, McDonnell C (2024). Barriers, facilitators, and recommendations to increase the use of a clinical decision support tool for managing chronic pain in primary care. Int J Med Inform.

[14] Kagarmanova A, Sparkman H, Laiteerapong N (2022). Improving the management of chronic pain, opioid use, and opioid use disorder in older adults: study protocol for I-COPE study. Trials.

[15] Elder JW, Wu EF, Chenoweth JA (2020). Emergency department screening for unhealthy alcohol and drug use with a brief tablet-based questionnaire. Emerg Med Int.

[16] Krebs EE, Gravely A, Nugent S (2018). Effect of opioid vs nonopioid medications on pain-related function in patients with chronic back pain or hip or knee osteoarthritis pain: the SPACE Randomized Clinical Trial. JAMA.

[17] Löwe B, Kroenke K, Gräfe K (2005). Detecting and monitoring depression with a two-item questionnaire (PHQ-2). J Psychosom Res.

[18] Damschroder LJ, Reardon CM, Widerquist MAO, Lowery J (2022). The updated Consolidated Framework for Implementation Research based on user feedback. Implement Sci.

[19] King N, Cassell C, Symon G Essential Guide to Qualitative Methods in Organizational Research.

[20] Rindal DB, Pasumarthi DP, Thirumalai V (2023). Clinical decision support to reduce opioid prescriptions for dental extractions using SMART on FHIR: implementation report. JMIR Med Inform.

[21] Dobscha SK, Corson K, Perrin NA (2009). Collaborative care for chronic pain in primary care: a cluster randomized trial. JAMA.

[22] Kroenke K, Krebs EE, Wu J, Yu Z, Chumbler NR, Bair MJ (2014). Telecare collaborative management of chronic pain in primary care: a randomized clinical trial. JAMA.

[23] Buntin MB, Burke MF, Hoaglin MC, Blumenthal D (2011). The benefits of health information technology: a review of the recent literature shows predominantly positive results. Health Aff (Millwood).

[24] Granviken F, Meisingset I, Bach K (2025). Personalised decision support in the management of patients with musculoskeletal pain in primary physiotherapy care: a cluster randomised controlled trial (the SupportPrim project). Pain.

[25] Garcia Reyes EP, Kelly R, Buchanan G, Waycott J (2023). Understanding older adults’ experiences with technologies for health self-management: interview study. JMIR Aging.

[26] Azad CL, Beres LK, Wu AW, Fong A, Giladi AM (2024). Developing a multimedia patient-reported outcomes measure for low literacy patients with a human-centered design approach. PLoS One.

