# Supplementary material for: Primary Care Clinician Perspectives on Older Adult Chronic Pain Management and Clinical Decision Support: Qualitative Study
Source: JMIR Form Res. 2025 Aug 26;9:e74381. doi: 10.2196/74381 (PMC12439314; doi:10.2196/74381)

**Appendix 1.** Improving Chicago Older Adult Opioid and Pain Management Through Patient-centered Clinical Decision Support and Project ECHO ® (I-COPE) Patient Pre-Visit Questionnaire

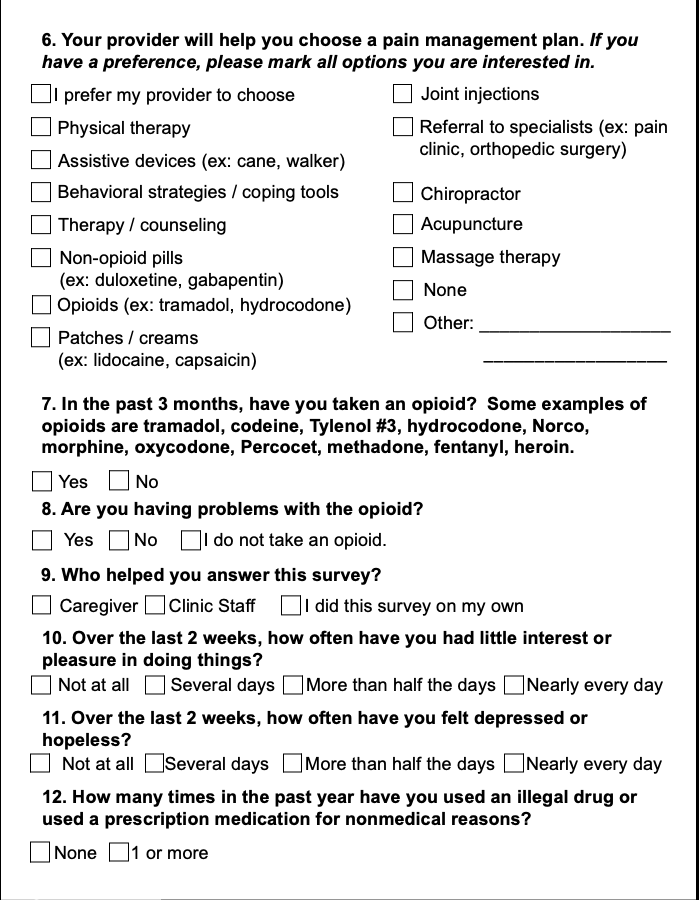

Supplement: Multimedia Appendix 1 [file formative-v9-e74381-s001.docx]
