# Supplementary material for: Primary Care Clinician Perspectives on Older Adult Chronic Pain Management and Clinical Decision Support: Qualitative Study
Source: JMIR Form Res. 2025 Aug 26;9:e74381. doi: 10.2196/74381 (PMC12439314; doi:10.2196/74381)
Supplement: Multimedia Appendix 2 [file formative-v9-e74381-s002.docx]

**Appendix 2.** Improving Chicago Older Adult Opioid and Pain Management Through Patient-centered Clinical Decision Support and Project ECHO ® (I-COPE) Electronic order set

1. **Collapsed**

**
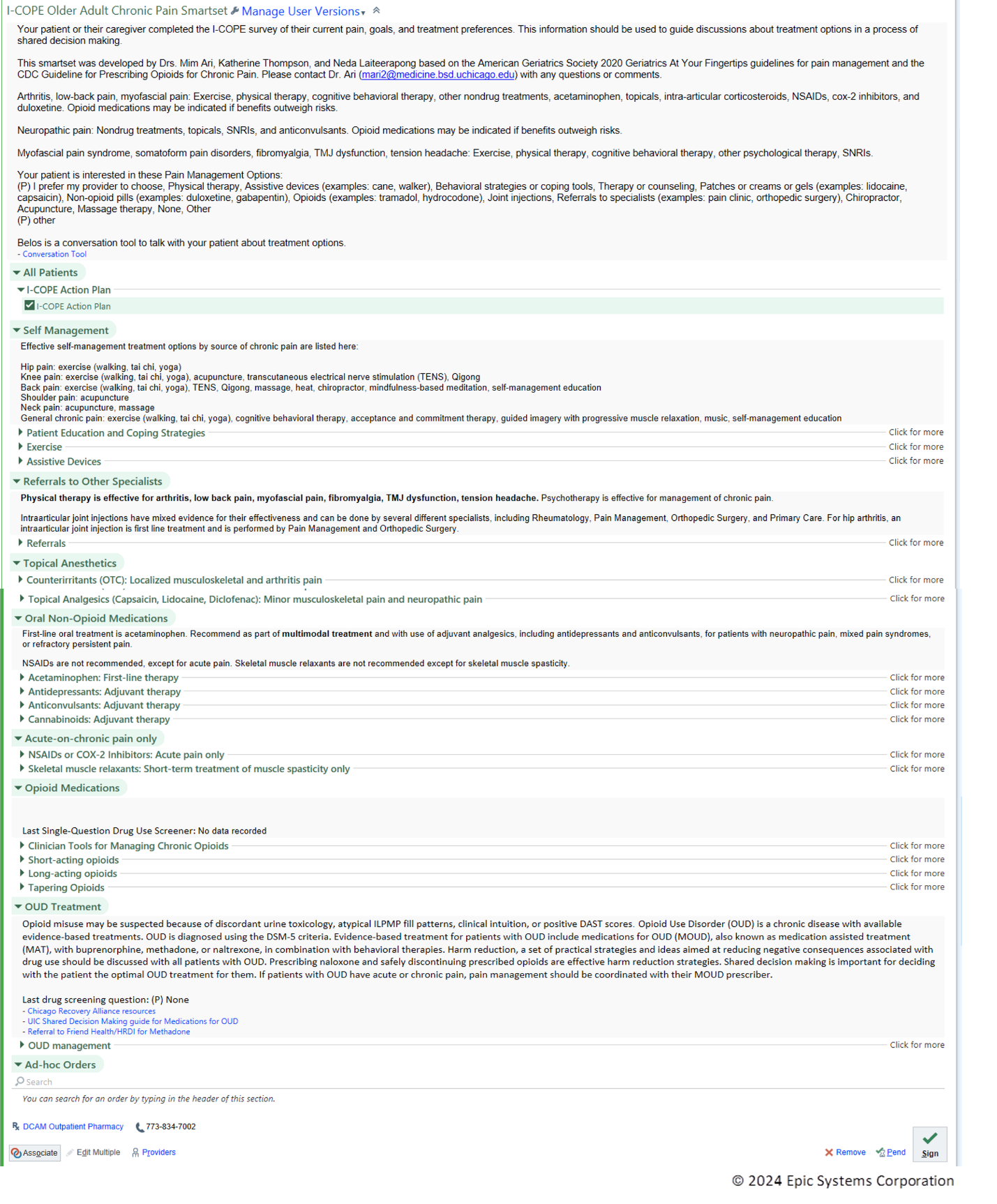
**

1. **Expanded**

**
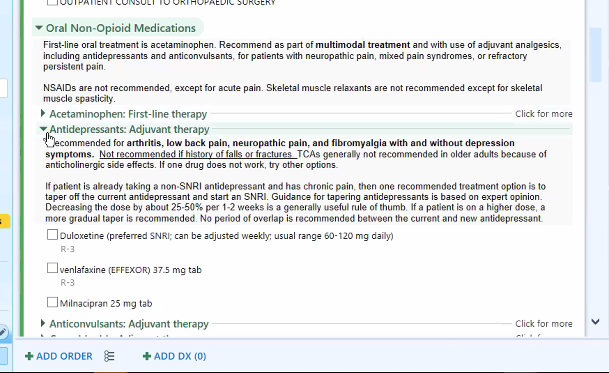
**

**
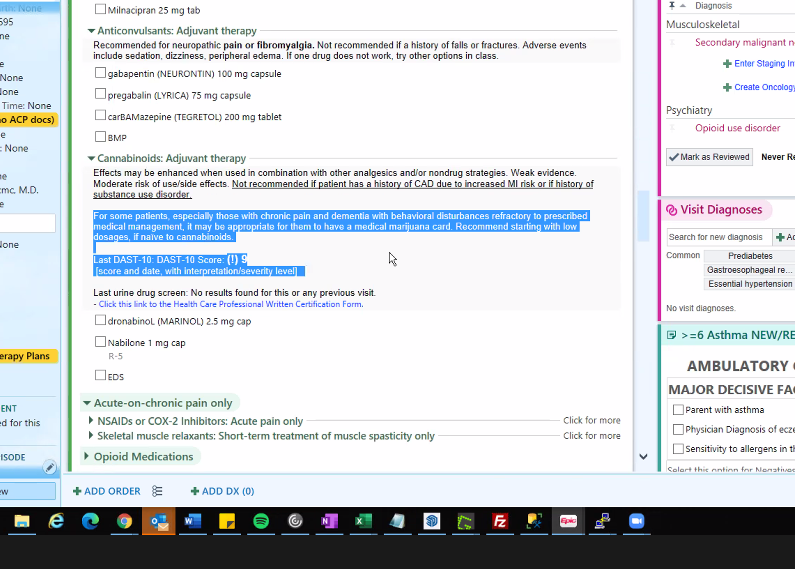
**

**
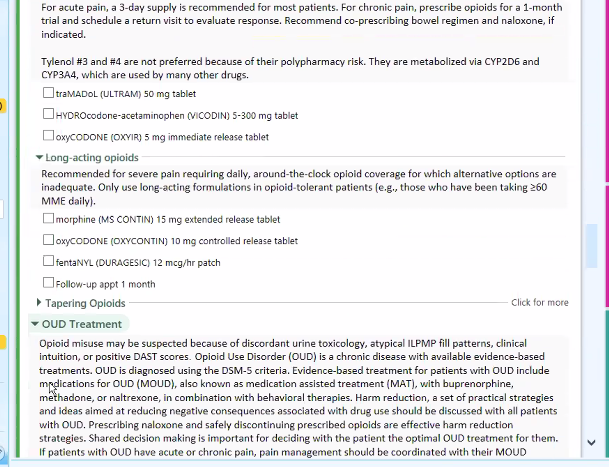
**

**
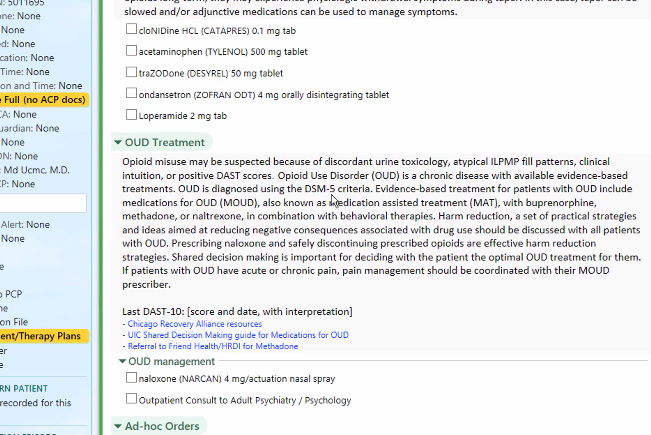
**

**
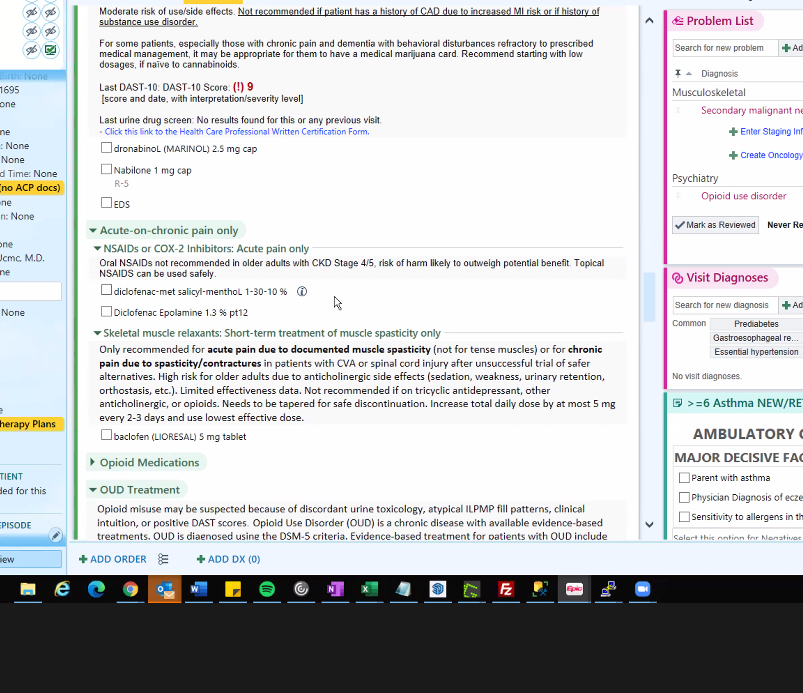
**
