# Supplementary material for: Primary Care Clinician Perspectives on Older Adult Chronic Pain Management and Clinical Decision Support: Qualitative Study
Source: JMIR Form Res. 2025 Aug 26;9:e74381. doi: 10.2196/74381 (PMC12439314; doi:10.2196/74381)

**Appendix 3.** Improving Chicago Older Adult Opioid and Pain Management Through Patient-centered Clinical Decision Support and Project ECHO® (I-COPE) Conversation tool


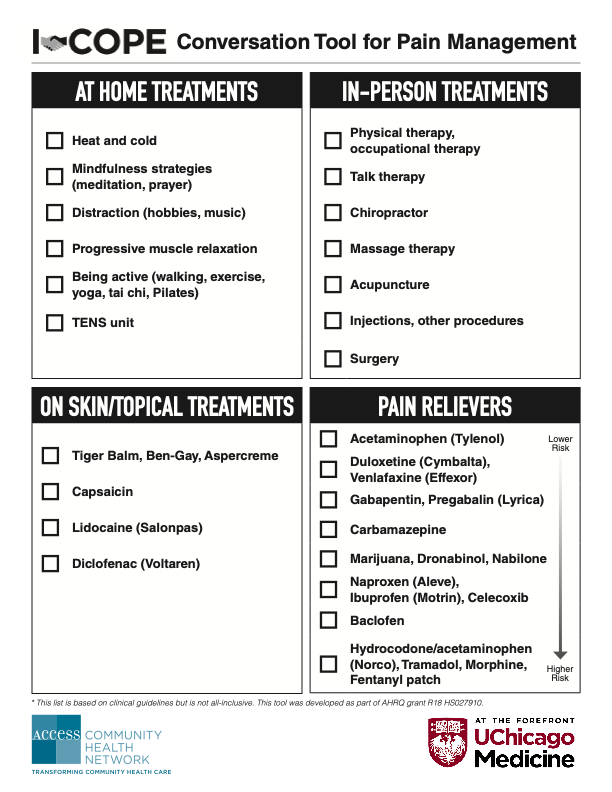

Supplement: Multimedia Appendix 3 [file formative-v9-e74381-s003.docx]
