# Supplementary material for: Primary Care Clinician Perspectives on Older Adult Chronic Pain Management and Clinical Decision Support: Qualitative Study
Source: JMIR Form Res. 2025 Aug 26;9:e74381. doi: 10.2196/74381 (PMC12439314; doi:10.2196/74381)
Supplement: Multimedia Appendix 5 [file formative-v9-e74381-s005.docx]

**Appendix 5**. I-COPE Primary Care Clinicians Interview guide

**Intro and Consent Script**

My name is Isra Hasnain. I work at the University of Chicago with Dr. Neda Laiteerapong, Dr. Kate Thompson, and Dr. Mim Ari, physicians in the Primary Care Group. Thank you for agreeing to participate in this interview. We are hoping to better understand the perspectives of primary care providers on the management of chronic pain, opioid use, and opioid use disorder in older adults, and to get feedback on the components of the ICOPE program that is currently being piloted at UCM. Your insight will help us understand what is working well and what could use improvements. With your oral consent, this interview audio will be recorded and then transcribed so we do not miss any comments, but all of your personal information will be eliminated from the recording and transcription. You can choose to not answer any questions and your participation is completely voluntary. Your name will not be linked with your comments. All information will be kept confidential. Do I have your permission to start this interview and begin recording?

Before we jump into the interview, I wanted to ask a few demographic questions; could I ask what race and gender you identify as? How many years have you been in practice?

First, I’ll be asking you about your current practices surrounding the management of chronic pain. Then, I will be presenting you with the individual I-COPE program components and asking for your feedback. Any questions before we start?

**General Questions**

As a provider, what are your thoughts on managing chronic pain in general? What about specifically among older adult patients?

What do you find challenging about managing chronic pain in older adults?

What are your thoughts on the strength of evidence for managing chronic pain?

What barriers do you have in following chronic pain guidelines?

What system-level barriers have you observed among patients trying to manage their chronic pain? (i.e., hindered access to physical therapists, insufficient knowledge)

Do you prescribe opioids for chronic pain? What patient factors do you consider when deciding to prescribe opioids for chronic pain? Do you consider patient age?

**Chronic pain and ICOPE questions**

How does your clinic currently assess pain?

What do you think about this system (assessing pain scores as part of the vital signs)? What do you like about this system? What do you not like about it? Are there other questions about chronic pain that would be helpful to ask?

How do *you* assess pain in your patients?

How is this process the same or different for chronic pain? How is this process the same or different for older adults?

How do you currently decide a patient’s chronic pain treatment plan? Do you ask about treatment goals? How?

You may know about a tool that is being incorporated into clinical practice known as the Pain, Enjoyment, and General Activity, or PEG, scale, which is a 3-item questionnaire in which patients are asked to rate on a scale of 0-10 the extent of their pain, to what degree pain interferes with their enjoyment of life, and how much pain interferes with their general activity. The I-COPE program will be using this questionnaire.

What do you think about these questions? Would this information be helpful to your clinical practice? Why or why not?

In the ICOPE program, there is a chronic pain questionnaire that is to be completed by patients prior to their appointment. Eligible patients are older adults with scheduled visits who have a chronic pain diagnosis, a recent pain score of 7 or higher, are taking opioid medications, and/or have opioid use disorder. They will be prompted to complete this questionnaire via an eVisit check-in on MyChart; or if they haven’t completed it by the time they’ve checked in, they will complete the survey at the clinic on an iPad or on paper. The questionnaire includes the PEG, scale, as well as a question about functional goals and preferred treatments. There is also a screening for depression and substance use disorder. The results are stored in Epic, and the survey results will be provided to you as a Best Practice Advisory. The Best Practice Advisory (or BPA) is non-interruptive and links to the ICOPE smartset.

I want to ask you for your thoughts about this system. How useful or not useful would having this system be? What do you think about the workflow? What are some challenges you anticipate? What improvements do you suggest?

How do you currently provide patient education on chronic pain?

How do you currently educate patients on chronic pain self-management? What resources do you provide to patients? What do you find challenging about patient education in this area?

Now, I’m going to show you the ICOPE smartset, which includes evidence-based treatments for chronic pain in older adults and guidance on when to use which treatments. The smartset has four major sections for chronic pain management and a section on opioid use disorder management. These sections include self-management patient education resources, topical treatments, non-opioid medications, and opioid medications.

Here is a list of the patient education resources provided in the I-COPE smartset and a sample of the instructions on exercises for knee pain. When an item is checked off, the content for that topic automatically goes into the after-visit summary patient instructions.

What are your thoughts on these types of educational materials? How are they helpful or not helpful?

We have integrated this list of treatment options [SHOW LIST], which is supported by the American Geriatric Society care guidelines. These include in-person treatments, at-home treatments, skin/topical treatments, and pain relievers.

In your current practice, are there any of these treatments that you do not routinely recommend to manage chronic pain? Why do you not recommend these treatments?

What are your thoughts on presenting treatment options in this list format? How is this helpful or not helpful?

**Opioid use disorder**

How do you currently assess for opioid use disorder? How is this process the same or different for older adults?

[Show Smartset components of ICOPE] In your current practice, what treatments or referrals do you routinely recommend to manage opioid use disorder? Why do you recommend these treatments?

Are there other evidence-based treatments that you routinely recommend? If so, why?

As part of ICOPE, we have also designed a conversational tool to help providers and patients navigate discussions about chronic pain treatment options.

What are your thoughts on providing this conversation tool in the clinic rooms? How useful or not useful would this be? What are some challenges you anticipate?

The final ICOPE program component is an action plan that revisits the patient’s average pain score, pain treatments, and patient education materials. This plan is automatically generated by what’s selected in the ICOPE smartset and not created by the provider. The plan outlines actionable strategies for the patient’s pain, which can include self-management, referrals, and/or medications.

What are your thoughts on developing an action plan as part of the ICOPE intervention? How useful or not useful would this be? What are some challenges you anticipate?

**Closing**

In our discussion today, I’ve mentioned the following ICOPE program components; chronic pain questionnaire, patient education and conversation tools, Smartset treatment options, and action plan. I want to get a feeling for your impressions on how this intervention as a whole may impact the care you provide for your patients.

What benefits do you see in this intervention?

How would this intervention be useful or not useful in your clinic? And for you and your patients? What challenges do you foresee regarding the use of this intervention as a whole?

Would you like the PI to reach out to you with clarifications about the questions you brought up?
