# Supplementary material for: Primary Care Clinician Perspectives on Older Adult Chronic Pain Management and Clinical Decision Support: Qualitative Study
Source: JMIR Form Res. 2025 Aug 26;9:e74381. doi: 10.2196/74381 (PMC12439314; doi:10.2196/74381)
Supplement: Multimedia Appendix 6 [file formative-v9-e74381-s006.docx]

**Appendix 6.** Anticipated Benefits and Challenges of Using I-COPE Intervention Components

| Anticipated Benefits | Anticipated Challenges |
| --- | --- |
| Intervention as a Whole | |
| “It's a nice way to ensure that there's just more support for non-opiate therapies,” Participant 6  “Just having more structure in place, I think will help a lot of people feel more comfortable about managing chronic pain,” Participant 1  “When there's more subjectivity in implementation patients get concerned that they're being discriminated against… If there is a systematic way we could do things such that patients know that this is the clinic's policy and this is how everybody does it here, there'll be less of that obstruction in the patient-provider relationship,” Participant 2 | “I think that lack of completeness, is going to be weird... if [patients] did the questionnaire, but then the doctor didn't talk about it…And then vice versa, if you didn't use the questionnaire, you're going to have a plan, without the assessment questions… is [the action plan] going to make sense to the patients when they take it home? If you didn't as a physician follow all the steps, and do it in order... Because I think, likely, people are going to use bits and pieces,” Participant 3  “Fully going into the SmartSet and running down the alternatives for it imagines a world where the pain diagnosis is the focus of the visit, and often it is not,” Participant 12 |
| Patient Pre-Visit Questionnaire | |
| “I think patients probably want to talk about pain more than we give them time to talk to about it, so it does provide a good way to get that down on paper… What I like about this survey is [it] lists all different kinds of pain management options… [and the question] about getting to their goals which is great,” Participant 4 | “Yeah, I think that it's definitely a good goal to have the patients do it before they go into clinic. But it's not really that realistic. A lot of the patients don't check their [patient portal], they don't even know how to access their [patient portal],” Participant 13 |
| Conversation Tool | |
| “I think it's nice to have those tools to go over with patients in the room as you're talking about options and all the different things that they can do to empower them,” Participant 18  “It would be well-suited for focusing on one of those boxes so if they're like, “What can I do at home?’ Or ‘I don't want to take a pill, what are the topical options?’ And that's a nice way to present it,” Participant 5 | “I think if the visit is there with the main priority of talking about pain, then I think this is a helpful adjunct. If there are multiple things to cover in a brief visit, then I feel like it may end up being more efficient to just discuss the most likely best fit treatments for the patient directly,” Participant 6  “I would like to have one that doesn't have [opioids] on it and one that does have [opioids] on it… when you've already had the discussion about it's not really indicated for your type of pain, it just brings that up again,” Participant 18 |
| Smart Order Set | |
| “I think being able to see everything on one screen makes it a lot easier for people to just go down and making sure they're hitting all the different multimodal analgesics.” Participant 8  “Having all that stuff easily accessible within a smart set, it's always nice, increase my likelihood talking about it. Then patients having the follow up of seeing it on their after-visit summary, so hopefully help solidify our conversation a little bit more,” Participant 9 | “In terms of the SmartSet, it's a lot of text. I think it would take some getting used to… it is hard because I also like to not use the computer during a patient visit. I tend to bring pen and paper so that I can be looking at the patient and not looking at the screen,” Participant 2 |
